# The brake matters: Hyperexcitable arousal circuits in sleep fragmentation with age

**DOI:** 10.1002/ctm2.900

**Published:** 2022-06-13

**Authors:** Shi‐Bin Li, Luis de Lecea

**Affiliations:** ^1^ Department of Psychiatry and Behavioral Sciences Stanford University School of Medicine Stanford California USA; ^2^ Wu Tsai Neurosciences Institute Stanford University Stanford California USA

**Keywords:** aging, sleep fragmentation, hyperexcitability, hypocretin/orexin neuron, neurodegenerative diseases

High‐quality sleep is essential for maintaining our physical and mental health. However, sleep quality declines with age. Aging not only brings daytime sleepiness, difficulty falling asleep and early awakenings, it also introduces conspicuous sleep fragmentation, which is the most common reason preventing the elderly from getting a restorative sleep.^1^ Despite the broad awareness of its high prevalence and detrimental effect on the body, the mechanistic underpinnings of sleep instability have been underexplored. Recently, we have studied why the brain loses its control of consolidated sleep during aging with a focus on investigation of arousal‐promoting neural circuits.^2^

We first hypothesized that the decline of sleep quality with age could be due to dysfunctional brain arousal circuits. Among these circuits, neurons producing the neuropeptide hypocretin^3^ (Hcrt, also known as orexin^4^) were very strong candidates with an established role in initiating and maintaining proper wakefulness.^5^, ^6^, ^7^, ^8^ Using laboratory mice, we found a significant loss of Hcrt neurons in the aged group, which exhibited a more fragmented sleep pattern. By recording calcium signals using fibre photometry, we found that aged Hcrt neurons displayed a higher frequency of Hcrt neuronal GCaMP6f activity driving more frequent wake bouts. The increase in Hcrt activity amplitude necessary to generate a successful sleep‐to‐wake transition was smaller in old mice compared to the young group, demonstrating a lower threshold for Hcrt‐induced awakenings in older mice (Figure 1A). Even though the aged group harboured fewer Hcrt neurons, optogenetic stimulation of these neurons elicited longer wake bouts compared with the young group using the same stimulation paradigm. Our in vivo optogenetic data indicated that the threshold of Hcrt neuronal activity defining sleep‐to‐wake transition is lower in aged mice, consistent with our calcium recording of spontaneous Hcrt activity. These data, accumulated with top‐down and bottom‐up strategies collectively support the hypothesis that emerging hyperexcitability of Hcrt neurons drives sleep fragmentation arising with age.

**FIGURE 1:**
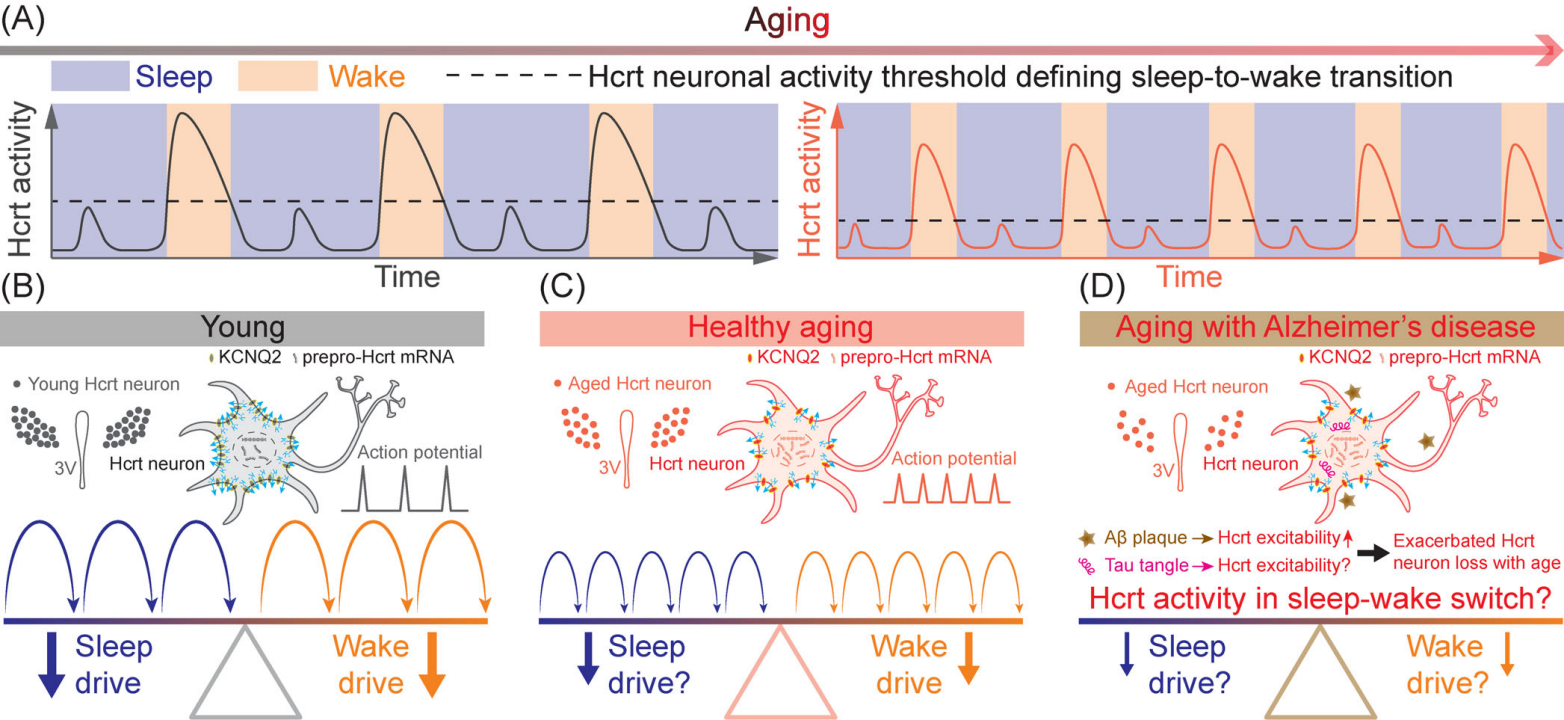
Emerging hyperexcitability of Hcrt neurons drives sleep instability with age. (A) More frequent Hcrt activities initiate more recurrent wake bouts with a lower Hcrt neuronal activity threshold defining sleep‐to‐wake transition in the aged group. (B) Hcrt neuron population, KCNQ2 expression density, firing pattern and sleep/wake drive in the young group. (C) Reduced Hcrt neuron counts, decreased KCNQ2 expression density with a more frequent firing pattern, and a more frequent, less robust sleep/wake drive with heathy aging. (D) A more severe Hcrt neuron loss in Alzheimer's disease with extracellular amyloid‐β plaque deposition and intracellular tau tangle accumulation. Figure was adapted with permission from Li et al.^2^

To unveil the cause of increased spontaneous Hcrt neuronal activity and a higher efficiency of Hcrt neuron activation in driving wakefulness in aged mice, patch‐clamp experiments were performed to determine the difference in electrophysiological properties between young and aged Hcrt neurons. The resting membrane potential (RMP) of aged Hcrt neurons was found to be depolarized, priming them to fire action potentials easier with smaller excitatory inputs. Light train optogenetic stimulation or current injection in aged Hcrt neurons expressing light‐sensitive ChR2 showed more spikelet activities, further validating our hypothesis.

A successful action potential is initiated with a depolarization sufficiently propelling the membrane potential to reach the firing threshold, then repolarization follows its peak appearance, and eventually the membrane potential reaches the resting state again.^9^ Considering the re‐polarization process functions as a “brake” system of a neuron's activity, we hypothesized that potassium (K^+^) re‐polarization currents are impaired with age. Voltage‐gated potassium channels (KCNQs) allow K^+^ to efflux from the intracellular to extracellular space, playing a critical role in the repolarizing phase of an action potential and maintaining the RMP.^10^ Recording of the M‐current, mediated by KCNQ2/3 channels, revealed a smaller M‐current amplitude in aged Hcrt neurons confirming a potential impairment of re‐polarizing ion channels in aged Hcrt neurons. Array tomography at ultra‐resolution demonstrated that the expression density of KCNQ2 was lower in aged Hcrt neurons, therefore, further corroborating the impaired M‐current from an anatomical perspective (Figure 1B and C). Single‐nucleus RNA sequencing revealed a lower fraction of Hcrt nuclei actively expressing the dominant sub‐types *Kcnq1/2/3/5* mRNAs, which is expected to contribute to the hyper‐excitability of aged Hcrt neurons. With the gene editing technique CRISPR/SaCas9, we disrupted a component of the “brake” system by targeting the *Kcnq2/3* genes in young Hcrt neurons, and found sleep fragmentation in young mice, recapitulating the instability feature of sleep in aged mice. Augmentation of the “brake” system with pharmacological activation of KCNQ2/3 channels re‐polarized the membrane potential, suppressed firing activity in aged Hcrt neurons and consolidated sleep bouts in aged mice. Expression level of prepro‐*Hcrt* mRNA doubled in aged mice compared to the younger cohort indicating a potential compensatory mechanism of Hcrt neuron loss in older mice.

To further explore the relevance of Hcrt neurons with emerging hyperexcitability in a neural network with age, we recorded local non‐fluorescent neurons intermingled with Hcrt neurons expressing ChR2‐eYFP in the lateral hypothalamus. Recording from neurons postsynaptic to Hcrt neurons expressing ChR2 demonstrated a higher successful ratio of optogenetic stimulation time‐locked post‐synaptic currents in the aged group. Among the downstream brain nuclei directly innervated by the Hcrt neurons, aged noradrenergic (NA) neurons in the locus coeruleus (LC) exhibited a trend of RMP depolarization and a higher fraction of recorded neurons with spontaneous firing activity, indicating a similar change to aged Hcrt neurons, in other wake‐promoting brain nuclei in aged mice. From a neural network perspective, these results collectively support our hypothesis that a lower sleep‐to‐wake transition threshold is defined for wake‐promoting brain nuclei in the aged brain.

Aging with neurodegenerative diseases, including Alzheimer's disease (AD), may exacerbate sleep disruption in the elderly population. In AD patients with mild cognitive impairment, increased Hcrt1 (Orexin‐A) levels in cerebrospinal fluid are associated with rapid eye movement sleep disruption and sleep fragmentation^11^ suggesting a potential upregulation of Hcrt neuronal activity with AD pathology. In APPswe/PS1δE9 (APP/PS1) AD mouse model, sleep quality markedly deteriorates with gradual amyloid‐β (Aβ) plaque deposition in the brain.^12^ Aβ is a factor known to hyperactivate hippocampal neurons via suppression of glutamate reuptake.^13^ Moreover, Aβ fragment Aβ_25‐35_ downregulates gene expression of *Kcnq2/3* and *Girk2/3/4* encoding KCNQ2/3 and G protein‐coupled inwardly rectifying potassium channels 2/3/4 (GIRK2/3/4), respectively, in hippocampal slices.^14^ Therefore, Aβ may upregulate neuronal activity through multiple mechanisms. In the context of tau pathology of AD, neuronal activity could be decreased, increased or remain unchanged depending on tau models, background strains, concentrations of tau and age‐related effects.^15^ Notably, Aβ deposition appears to accelerate tau spreading and cognition impairment in AD patients.^15^ In parallel, sleep deterioration may generate a vicious cycle with Aβ deposition and tau development in patients with AD. A more severe neuronal loss of several arousal‐promoting brain nuclei, including Hcrt neurons, LC NA neurons, tuberomamillary nucleus (TMN) histaminergic neurons, in post‐mortem brains of AD patients with tauopathy has been observed.^16^ In a longitudinal clinical cohort study, more surviving Hcrt and TMN neurons correlated with decreased homeostatic sleep drive, shorter sleep bout duration, and greater percentage of wakefulness after sleep onset,^17^ indicating an upregulation of excitability in these neurons. The glymphatic system promotes movement of cerebrospinal fluid into the brain and clears metabolic waste with a more efficient clearance during sleep.^18^ Therefore, enhancing the “brake” system of these wake‐promoting neurons may suppress their hyperexcitability, consolidate sleep continuity, boost the glymphatic system and counteract the progression of AD pathology.

Our work supports the hypothesis that a more frequent, less robust wake drive with a lower sleep‐to‐wake transition threshold causing sleep fragmentation emerges with age. The upregulated excitability of Hcrt neurons is associated with an impaired “brake” system including the reduction of the repolarizing M‐current. The loss of K^+^ channels during aging may be due to oxidation at the protein level^19^ and a reduction of gene transcription, as we recently demonstrated.^2^ AD dramatically influences arousal‐promoting brain nuclei including Hcrt neurons. Patients with AD manifest both extracellular Aβ plaques and intracellular tau‐containing neurofibrillary tangles in the brain.^15^ Based on our understanding, it is likely that development of Aβ pathology will further elevate Hcrt neuronal activity, which will inevitably introduce excitotoxicity leading to neuronal death in the elderly with AD. Electrophysiological interrogation of Hcrt neurons with exposure to Aβ pathology will greatly advance the understanding toward this avenue. Follow‐up experiments may be conducted to tease apart the roles of extracellular Aβ plaques and intracellular tau‐containing neurofibrillary tangles in affecting wake‐promoting brain nuclei including Hcrt neurons (Figure 1D). On the other side, a study based on human data suggests a potential change of sleep‐promoting arousal circuits, including galaninergic neurons, during aging and with AD.^20^ Further investigation of the mechanisms underlying alterations to sleep‐promoting neural circuits during healthy aging and with neurodegenerative diseases may lead to more accurate and personalized treatments for these disorders.

## CONFLICT OF INTEREST

The authors declare no conflict of interest.
